# Limb Regeneration in *Xenopus laevis* Froglet

**DOI:** 10.1100/tsw.2006.325

**Published:** 2006-05-12

**Authors:** Makoto Suzuki, Nayuta Yakushiji, Yasuaki Nakada, Akira Satoh, Hiroyuki Ide, Koji Tamura

**Affiliations:** Department of Developmental Biology and Neurosciences, Graduate School of Life Sciences, Tohoku University, Aobayama Aoba-ku, Sendai 980-8578, Japan

**Keywords:** epimorphosis, limb regeneration, dedifferentiation, blastema, spike, nerve dependence, muscle regeneration, wound healing

## Abstract

Limb regeneration in amphibians is a representative process of epimorphosis. This type of organ regeneration, in which a mass of undifferentiated cells referred to as the “blastema” proliferate to restore the lost part of the amputated organ, is distinct from morphallaxis as observed, for instance, in Hydra, in which rearrangement of pre-existing cells and tissues mainly contribute to regeneration. In contrast to complete limb regeneration in urodele amphibians, limb regeneration in *Xenopus*, an anuran amphibian, is restricted. In this review of some aspects regarding adult limb regeneration in *Xenopus laevis*, we suggest that limb regeneration in adult *Xenopus*, which is pattern/tissue deficient, also represents epimorphosis.

## PROCESS OF LIMB REGENERATION IN VERTEBRATES

Regenerative ability of appendages (limbs/fins) in vertebrates varies greatly[1]. Teleost fishes are capable of regenerating radial rays of their pectoral and pelvic fins as well as caudal fins, but they cannot regenerate internal skeletal elements at the base of their fins[2,3]. Birds such as chickens cannot regenerate even limb buds at any stage of development, though the implantation of additional AER (apical ectodermal ridge)- or FGF-soaked beads partially rescues the limb structure of amputated limb buds[4,5,6]. Some ability for limb regeneration has also been reported in mammals. Neonatal mice as well as embryos can regenerate their digit tips[7,8], and this digit tip regeneration also occurs occasionally in humans[9,10]. Although these observations indicate the possibility of therapy-induced regeneration of limbs in mammals, including humans, the ability for limb regeneration in mammals is clearly poor.

In contrast, the potential for limb regeneration in amphibians, including urodeles such as the axolotl and newts in particular, is outstanding among vertebrates[11,12,13]. After amputation of a limb at any level, urodeles can completely regenerate the lost part of a limb throughout their life. This fascinating process of perfect limb regeneration in urodeles involves the following key steps (Fig. 1A), and the limb regeneration sequentially proceeds through these steps in a temporally overlapping and spatially coordinated manner.

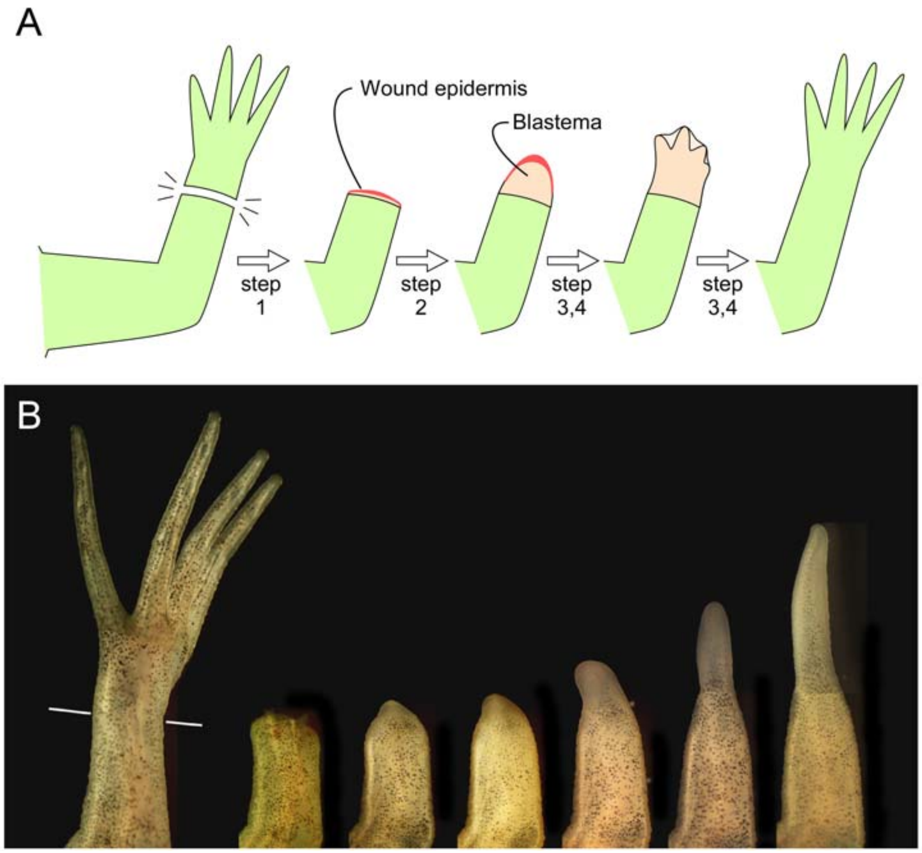

**FIGURE 1.** Limb regeneration in the urodele and *X. laevis* froglet. (A) A schematic representation of the process of limb regeneration in the urodele showing that the process can be divided into several overlapping steps. See text for details. (B) Spike formation in the *X. laevis* froglet forelimb. The right forelimb of a froglet (1.5–2 cm at snout-to-vent length) was amputated through the distal zeugopodium (indicated by a white line), and the amputation surface was trimmed to be flat. The animal was kept at 23–24°C in dechlorinated tap water. Photographs show the intact limb before amputation and the stump at 0, 7, 10, 14, 21, 28 days after amputation (left to right).

1. Within 1 day after limb amputation, the amputated surface is rapidly covered with epithelial cells following formation of a specialized dermis-free epithelial structure referred to as "wound epidermis". As the regeneration process progresses, this epithelial structure produces an apical epithelial cap (AEC), a structure that is morphologically and functionally similar to the AER. The AER emanates a number of growth factors in order to allow morphogenesis for limb bud outgrowth and patterning[14,15,16].
2. After the wound epidermis formation, mesoderm-derived tissues beneath the wound epidermis undergo histolysis triggered by matrix metalloproteinases (MMPs)[17,18], leading to the next important process, dedifferentiation, for generating a population of undifferentiated cells, including proliferative and multipotent mesenchymal cells. At the end of the dedifferentiation stage, the mesenchymal cells build up a "blastema", a cone-shaped mass of cells that is a structure comparable to the limb bud in limb development.
3. Until the process of limb regeneration is completed, the blastema continues to grow distally by active proliferation of blastemal mesenchymal cells. This process requires a close interaction between the mesenchyme and AEC, which is probably mediated by several growth factors[19,20,21,22], including FGFs as in limb bud elongation[14,15,16,23,24].
4. Simultaneously with blastema elongation, redifferentiation and repatterning begins, and a complete limb structure is finally re-established.

If a defect is caused by surgical operation, pharmacological treatment, or spontaneous accident at any step in the process, proper limb regeneration is prevented, resulting in the formation of a hypomorphic structure. Thus, examinations of various defects in the limb regeneration process and investigations at molecular and cellular levels are promising approaches in order to understand the nature of epimorphosis in vertebrates. Covering the amputated plane with fully mature skin flaps, for example, inhibits formation of the wound epidermis and blastema by preventing epithelial-mesenchymal interactions[25]. The function of the wound epidermis is partially mediated by MMPs elicited from the wound epidermis; the wound epidermis has been shown to produce MMP9[26], MMP3/10[27,28] and a collagenase (nCollagenase)[28], and a synthetic MMP inhibitor, GM6001, has been shown to induce malformation or scar formation in newt limb stumps[28].

## PROCESS OF LIMB REGENERATION IN *XENOPUS*

The South African clawed frog, *Xenopus laevis*, is an anuran amphibian in which limb regeneration has been studied in detail. This frog was named from its strange (xeno-) limbs (-pus), which have a claw in some anterior digits of the hindlimbs. The strange character of the limb in *Xenopus* is also true of its developmental stage-dependent ability of limb regeneration[29]. During tadpole stages before metamorphosis, they can regenerate their amputated limb buds perfectly. Their ability to fully regenerate a hindlimb bud continues until stage 52–53, early stages of limb development, but then gradually declines in terms of pattern formation. The failure in pattern formation results in incomplete regenerates, which have a smaller number of digits, 4 to 1 (*Xenopus* normally have 4 digits and 5 digits in their forelimbs and hindlimbs, respectively.). At the climax of metamorphosis (stage 56–57), when their hindlimbs have differentiated, well-organized muscle tissue and well-patterned skeleton, their hindlimb enters into a refractory period when amputated hindlimbs cannot regenerate any structures and amputation results in simple wound healing[30]. After the refractory period, when the tadpole has changed its morphology into a froglet, limb amputation gives rise to a regenerate, an unbranched long cartilaginous protrusion referred to as a “spike” (Fig. 1B)[31].

Bryant and colleagues examined the regenerative ability of *Xenopus* limbs by experiments in which various amounts of autopodial tissues were removed[32], or the tissues were exchanged between hindlimb buds of stage 52–53 tadpoles and froglet forelimb blastemas[33], and they found that the regenerative ability of a *Xenopus* limb is dependent on the developmental stage of the blastema rather than on environmental conditions supplied by the host. When developing hindlimb buds are grafted onto froglet limb stumps and amputated, they regenerate as they do on tadpole limbs, whereas froglet forelimb blastemas grafted onto tadpole hindlimb stumps cannot regenerate well-patterned digits but merely form spikes[33]. These findings suggest that the decline in regenerative ability is due to intrinsic properties of limb cells rather than extrinsic properties such as neurotrophic factors or growth hormones. These pioneering findings imply that there are some important differences between tadpole and froglet cells in *Xenopus* limb as a hint to resolve why some vertebrates such as humans cannot regenerate limbs, whereas others can. Therefore, many investigators have been fascinated to study mechanisms involved in limb regeneration of *Xenopus*.

There has been debate as to whether spike formation of the *Xenopus* froglet is based on simple tissue regeneration or epimorphosis. From histological observations, some researchers argued that spikes do not develop from the epimorphic blastema containing dedifferentiated mesenchymal cells, but only from proliferative differentiated cells such as fibroblasts and chondrocytes within connective or skeletal tissues[34,35]. On the other hand, Goss and Holt[36] investigated whether spike formation normally occurs if naked (skinless) *Xenopus* limb stumps were inserted into the abdominal cavity. In the case of urodele limbs, the insertion of naked limb stumps results in the formation of neither wound epidermis nor blastemas, but in the formation of cartilaginous caps reminiscent of typical tissue regeneration in vertebrates. As expected, most of the inserted *Xenopus* limb stumps could not form spikes, but resulted in cartilaginous caps, suggesting that *Xenopus* spike formation requires the wound epidermis and the blastema, both of which are only formed in epimorphosis. Thus Goss and Holt assumed that spike formation in the *Xenopus* froglet occurs on epimorphosis conducted by wound epidermis and dedifferentiated blastemas. Recent studies using newly available molecular approaches have provided more evidence supporting this idea. In particular, nerve dependency of *Xenopus* spike formation described in the following section strongly suggests that *Xenopus* limb regeneration is epimorphic.

## DEDIFFERENTIATION AND NERVE DEPENDENCE IN THE *XENOPUS* LIMB REGENERATION

Fig. 2 shows a series of histological sections of *Xenopus* froglet (1.5–2 cm in snout-to-vent length) forelimb stumps at 0–4 days after amputation. Immediately after limb amputation, bone began to protrude from the amputation plane because of contraction of other soft tissues such as skin, connective tissue, and muscle (Fig. 2A). Within 1 day after limb amputation, wound epidermis had covered the amputation plane to isolate it from the external environment (Fig. 2B). In this period, morphological changes, including histolysis, were not detected and internal mature tissues were still in direct contact with the overlying epidermis. Regardless of unchanged morphology, however, progenitors of blastemal mesenchymal cells may have already begun to be stimulated in response to limb amputation because BrdU incorporation into the nuclei of cells in internal tissues increased within 1 day (Fig. 2E,F). Within another day, the number of BrdU-labeled cells remarkably increased (Fig. 2G) and internal soft tissues were collapsed by histolysis under the wound epidermis to generate a space for accumulation of progenitors of blastemal mesenchymal cells (Fig. 2C). Simultaneously, the wound epidermis became thickened, but the number of BrdU-labeled epithelial basal cells in the wound epidermis decreased. This reduction of proliferating cells in the wound epidermis was transient, and these cells actively proliferated again at 7 days after amputation[37]. At 4 days after amputation, BrdU-labeled cells, which are morphologically undifferentiated cells, had preferentially accumulated in the space beneath the wound epidermis (Fig. 2H). In addition, the periosteum attached to residual protruding bones in the center of this space had started to be thickened. Finally, at about 4 days after amputation, an early-stage blastema was established (Fig. 2D). These detailed observations revealed that these sequential events during the early stage of spike formation in *Xenopus* froglet are comparable with those in limb regeneration of urodeles[38,39], though the time schedules of the events differ depending on body size[40]. It is noteworthy that some of the early events in the first step of limb regeneration, including blastema outgrowth, require axons and neurotrophic factors that the axons produce in both urodeles and anurans, and that other early events proceed nerve-independently[41,42].

Nerve dependency is a characteristic feature of epimorphic limb regeneration in amphibians. In urodeles, axons in the limb, which originate from spinal cord and dorsal root ganglia of the vertebral column, secrete neurotrophic factors into the blastema that are essential for its growth. Therefore, surgical removal of these axons inhibits proper development of the blastema and results in the simple restoration of wounded tissue[reviewed by 40]. These neurotrophic factors have been shown to mediate blastema formation as mitogenic[43,44,45] and survival[46] factors for dedifferentiated blastemal mesenchymal cells, and some growth factors, including transferrin, neuregulins (GGF), and FGFs, have been proposed as candidate molecules[47,48,49,50,51,52] that stimulate/maintain blastema outgrowth by regulating the expression of genes such as *Fgfs*[31,53,54]. Interestingly, denervation gives rise to ectopic dermis formation beneath the wound epidermis/AEC and reduction of the epithelial structure, suggesting that these factors play a role also in the formation of normal and active wound epidermis[55]. Although the importance of neurotrophic factors in limb regeneration has been widely accepted, the molecular mechanism of the nerve dependency remains unclear. Whether or not nerve dependency of blastema formation is also applicable to *Xenopus* species has been controversial because a previous study[56] showed that a denervation on a *Xenopus* limb failed to prevent the spike from being formed. However, Endo et al.[31] established an uprooting denervation method for *Xenopus* froglet limbs and found that this method is sufficient to inhibit spike formation at a high frequency as in urodele limbs. It is reasonable to

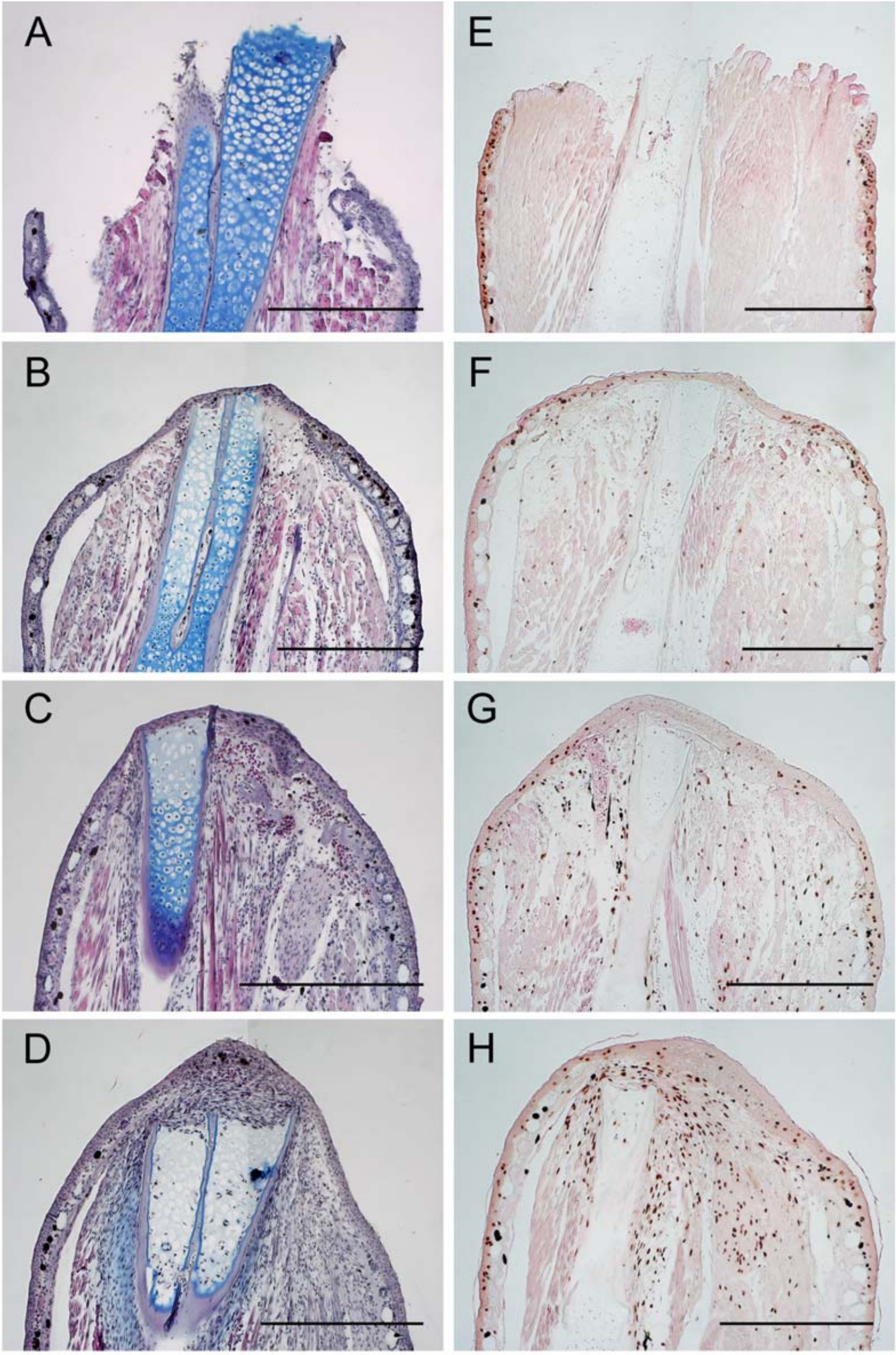

**FIGURE 2.** Histological observations of early limb blastema in the *Xenopus* froglet. Longitudinal sections of the froglet forelimb stumps were stained with hematoxylin, eosin and Alcian blue (A–D) or with anti-BrdU antibody and DAB (brown) after incorporation of BrdU for 1 h (E–H). (A,E) 0 days, (B,F) 1 day, (C,G) 2 days, (D,H) 4 days after amputation. Specimens were fixed in Bouin's fixative, embedded in paraffin, and sectioned at a thickness of 6 μm. The procedures for the BrdU treatment and immunohistochemistry were essentially the same as that of Suzuki et al.[37]. Scale bars = 400 μm.

conclude that nerve dependency is a key event for epimorphosis that is common to urodeles and anurans. Furthermore, our recent study[37] on the molecular framework of *Xenopus* froglet limb regeneration during the early phase (within 10 days postamputation) demonstrated similarities and differences among nerve-dependent and nerve-independent events. Histological examinations showed that neuronal effects on limb stumps begin by 7 days after amputation and that denervated limbs exhibit morphologies similar to lateral wound healing induced by skin removal. Consistent with these observations, numerous axons present at the proximal region of limb stumps invade beneath the established wound epidermis within a week after amputation, and these axons increase in number until 10–14 days after amputation (Fig. 3). These observations, together with results of BrdU incorporation and TUNEL assays, suggest that some events for dedifferentiation, including wound epidermis formation, histolysis, and re-entry into the cell cycle, could proceed without neuronal effects in the *Xenopus* froglet. Since the same conclusion has been obtained in urodeles[43,44,45,46], the characteristics of nerve dependency of *Xenopus* froglet limbs seem to be closely related with those of urodeles.

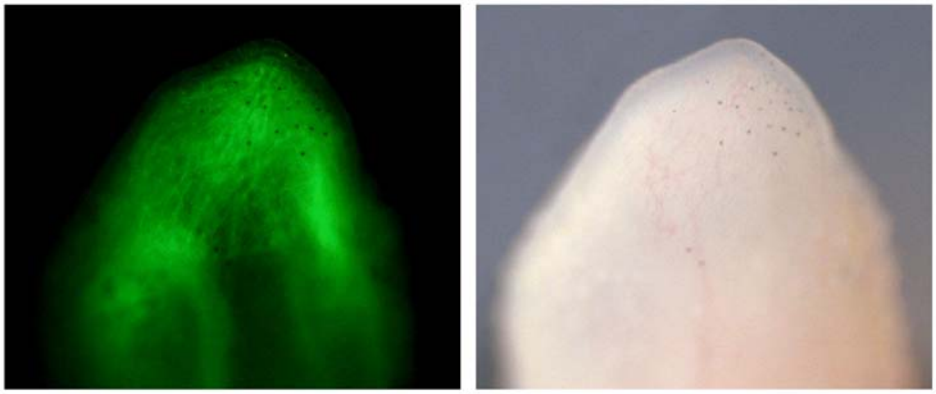

**FIGURE 3.** A ventral view of the right forelimb blastema of an N-tubulin promoter/GFP transgenic *X. laevis* froglet at 14 days after amputation. N-tubulin promoter directs reporter gene expression to the neurons[81], and it is noted that numerous GFP-expressing axons are distributed in the blastema. The right panel is a brightfield image of the left panel. Transgenesis was conducted following the procedure described by Kroll and Amaya[81], and the plasmid for transgenesis (N-tubulin promoter/GFP: NBTG) was a kind gift from Enrique Amaya (Gurdon Institute, Cambridge, U.K.)[81,82,83].

Some transcription factors and signaling molecules that play important roles in limb development[57], including *Msx1*, *Fgf8*, and *Fgf10*, are expressed in the growing blastema in a nerve-dependent manner[31,37]. Other molecules, including *Tbx5* and *Prx1*, accurate markers for initiation of a limb bud[58,59,60,61], have been shown to be up-regulated independently of nerve supply, but their expression cannot be maintained without nerve supply[37]. During limb development, *Tbx5* acts as an upstream regulator of *Fgf10*[62,63]. Moreover, during the refractory period, the nonregenerating *Xenopus* hindlimb can regenerate well-patterned structures and induce *Msx1*/*Fgf8/Fgf10* by exogenous application of FGF10[30,64]. Taken together with the fact that onset of *Tbx5*/*Prx1* re-expression in the blastema is prior to that of *Msx1*/*Fgf8/Fgf10*, it is possible that nerve-dependent maintenance of *Tbx5*/*Prx1* may yield *Msx1*/*Fgf8/Fgf10* expression to induce outgrowth of the blastema. Functions of these key molecules and identification of upstream regulators in these events are the next issue, and a combination of exhaustive investigation and functional analyses that have recently been available for *Xenopus* should contribute to a further understanding of the initial step of epimorphic limb regeneration. Interestingly, a lateral skin wound also expresses both *Tbx5* and *Prx1* genes[37], suggesting that the molecular characteristics of the early phase of limb regeneration are closely related with those of simple wound healing. Indeed, it has also been shown that both denervated blastemas and lateral skin wound failed to maintain the expression of these genes, supporting this idea.

## REPATTERNING OF AMPUTATED FROGLET LIMBS

Whereas urodeles can reconstruct an exact replica of the amputated limb, the *Xenopus* froglet cannot but regenerate a cartilaginous spike that has no skeletal pattern as described above. Histological observations showing that this hypomorphic structure has neither segmentation nor bifurcation in the cartilage suggest that froglet blastemas have some deficiencies in the ability for pattern formation.

Pattern formation of the limb has been extensively studied in the system of limb development in amniotes. This patterning involves morphogenesis along three axes (antero-posterior (AP), dorso-ventral (DV) and proximo-distal (PD) axes) (Fig. 4). Pattern formation along these axes is quite complex and several molecules have been shown to play key roles in the establishment of a well-organized skeletal pattern[57]. For example, *sonic hedgehog* (*shh*), a vertebrate homolog of *Drosophila hedgehog* that is expressed at the zone of polarizing activity (ZPA), regulates the AP axis and the number of digits in cooperation with *Gli3*, a multifunctional transcription factor[65,66]. For the other two axes, *Lmx1*, a homeobox-containing gene expressed in the dorsal mesenchyme of the limb bud, has been shown to be a main regulator of the DV axis[57], and some *Hox-a* cluster genes, including *Hoxa11* and *Hoxa13*, contribute to the PD axis[67,68]. To try to determine why froglet limb stumps form spikes, our previous studies focused on the expression pattern of *shh*[31] and *Lmx1*[69], and we found that neither of these genes are expressed in the froglet blastema, whereas both are appropriately re-expressed in the limb bud blastemas of *Xenopus* larvae (Fig. 4)[69,70]. Taken together with the fact that the expression pattern of these genes in urodele limb blastemas is similar to that in developing limb buds, these results suggest that patterning in limb regeneration is basically organized by the same molecular mechanisms as those in limb development, and that the deficient ability for pattern formation in the *Xenopus* froglet limb include defects of these molecular mechanisms. However, (1) the fact that the spike of a male frog can develop nuptial pad tissue on the ventral side that is required for amplexus and successful mating[71]; (2) the fact that *Hoxa13*, a marker of the prospective autopodium region, is expressed preferentially in the distal region of the froglet blastema[31]; and (3) the fact that some of the important genes regulating AP axis development are expressed also in the froglet blastema (N.Y., unpublished observation) suggest that some features along each axis are reconstructed. The ability for pattern formation in the froglet limb may not be absent, but insufficient for complete reconstruction.

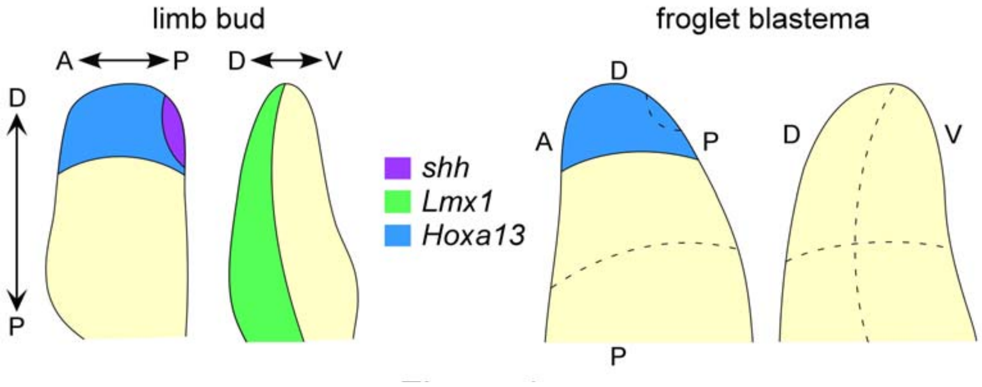

**FIGURE 4.** Schematic representations showing expression pattern of regulatory genes important for pattern formation along three axis (A–P, anterior-posterior; D–V, dorsal-ventral; P–D, proximal-distal) in the tadpole limb bud and froglet blastema. *Shh* (purple) is expressed in the posterior margin of the limb bud mesenchyme (ZPA). *Lmx1* (green) is expressed in the dorsal mesenchyme of the limb bud. However, neither of these genes is expressed in the froglet blastema. On the other hand, *Hoxa13* (blue) is expressed in the distal region of both the limb bud and froglet blastema.

## REDIFFERENTIATION OF AMPUTATED FROGLET LIMBS

Since pattern formation of the limb bud is accompanied and even influenced by tissue differentiation, temporally and spatially coordinated tissue differentiation should be important for successful regeneration of vertebrate limbs. Urodele limb blastemas can regenerate all tissue components, including epithelial tissues (skin glands), skeletal tissues (bone and cartilage), soft connective tissues (dermis, tendon and ligament), and muscles with satellite cells. In addition, proper angiogenesis[72] and innervation (see above) of the blastemas are required for proliferation and survival of the blastemal mesenchymal cells. On the other hand, the spike of the froglet shows a limited ability for tissue regeneration. Histological observations in *Xenopus* adult limb regeneration have revealed no muscle, mineralized bone, joint, tendon, and ligament, whereas skin gland formation occurs normally[31,73,74]. Regeneration of nuptial pad tissue[71] and a claw[75] in the spike, both of which are epidermal derivatives, suggests that the deficiency of tissue differentiation characteristically appears in mesodermal derivatives.

Recent approaches at the molecular level[73,74,76] have provided some new insights into the extent of defects and whether they could be improved by therapeutic treatment. Analysis of the expression of myogenesis-related marker genes and proteins, including PAX7, *MyoD*, and *Myf5*, revealed that neither differentiated muscle nor satellite cells exist in the spike[73]. However, implantation of purified muscle precursor cells or hepatocyte growth factor (HGF)–releasing cell aggregates induced muscle regeneration in the spike, indicating that the froglet blastema provides a permissive environment for survival and differentiation of myoblasts. It is possible that muscle precursor cells are absent from the blastema or, alternatively, that those precursor cells in the blastema have some defects for differentiation. Immunohistochemical analysis revealed that the cartilage of the *Xenopus* froglet spike lacks any joint and a mature perichondrium layer[76]. This deficiency was associated with the absence of correct expression of *bmp4* and *gdf5*, both of which are expressed in the perichondrium layer of the developing limb bud. Indeed, implantation of BMP4-soaked beads induced joint-like segmentations on the cartilage of the spike. Within these structures, nuclear localization of phosphorylated Smad-1/5/8 protein, a downstream component of the BMP-signaling pathway, was observed[76], suggesting that applied BMP4 induced the segmentations in a way similar to that reported in limb development[77,78]. Furthermore, froglet blastemas have been shown to express *dermo-1* and *scleraxis*, markers for dermal progenitor cells and tendon/ligament, respectively[74]. These findings suggest that blastemal mesenchymal cells possess the competence to differentiate into these tissues.

In summary, the froglet blastema is likely to contain many types of progenitor cells (epithelia, cartilage, blood vessels, dermis, and tendon/ligament cells), and at least two major deficiencies of the froglet spike (muscleless and jointless) are improvable by therapeutic treatments. Taken together with previous reports that mutual interaction between muscle and tendon cells is required for correct tendon development[79,80], simultaneous improvement of muscle and joint regeneration is perhaps necessary for reconstruction of functional limb-like structures on froglet limb stumps.

## PERSPECTIVE

In this article, we have reviewed old and recent information on *Xenopus* limb regeneration, and we would like to emphasize that limb regeneration in the *Xenopus* froglet represents epimorphosis and is comparable to that in urodele amphibians, although this process gives rise to an incomplete regenerate. Thus, this useful animal serves as a good model system to investigate epimorphic limb regeneration. The considerable accumulation of molecular information, sources, and techniques, including transgenesis in *Xenopus* in particular (as shown also in this review in Fig. 3), will enable many unsolved issues regarding limb regeneration to be addressed. Also, new approaches to improve the pattern-less spike to a complete limb structure will ultimately give hints to regenerate human limbs.

## ACKNOWLEDGMENTS

We are grateful to Dr. David Stocum for critical reading of this manuscript and comments on it. We thank Dr. Enrique Amaya for the kind gifts of the plasmid. Work in our laboratory is supported by research grants from the Ministry of Education, Science, Sports and Culture of Japan.
